# Oxidative DNA damage in the rat lung induced by intratracheal instillation and inhalation of nanoparticles

**DOI:** 10.3164/jcbn.17-70

**Published:** 2018-02-07

**Authors:** Yun-Shan Li, Yuko Ootsuyama, Yuya Kawasaki, Yasuo Morimoto, Toshiaki Higashi, Kazuaki Kawai

**Affiliations:** 1 Department of Environmental Oncology, Institute of Industrial Ecological Sciences, University of Occupational and Environmental Health, 1-1 Iseigaoka, Yahatanishi-ku, Kitakyushu 807-8555, Japan; 2 Department of Occupational Pneumology, Institute of Industrial Ecological Sciences, University of Occupational and Environmental Health, 1-1 Iseigaoka, Yahatanishi-ku, Kitakyushu 807-8555, Japan; 3 President, University of Occupational and Environmental Health, 1-1 Iseigaoka, Yahatanishi-ku, Kitakyushu 807-8555, Japan

**Keywords:** nanoparticle, 8-hydroxydeoxyguanosine (8-OHdG), oxidative DNA damage, intratracheal instillation, inhalation

## Abstract

Nanoparticles are widely used as useful industrial materials. Therefore, their possible adverse health effects must be appraised. We assessed and compared the oxidative DNA damage caused by four different nanoparticles (TiO_2_, NiO, ZnO and CeO_2_). The effects of the administration methods, intratracheal instillation and inhalation, were also evaluated. Rats were subjected to intratracheal instillations or 4 weeks of inhalation exposure to the nanoparticles, and the 8-hydroxydeoxyguanosine (8-OHdG) levels in the lung were analyzed by an HPLC-EC detector method. The 8-OHdG levels were increased in a dose-dependent manner with the inhalation of NiO. ZnO also increased the 8-OHdG levels with inhalation. In comparison with the control, the 8-OHdG levels were significantly and persistently higher with the CeO_2_ nanoparticle administration, by both intratracheal instillation and inhalation. In contrast, there were no significant differences in the 8-OHdG levels between the control and TiO_2_ nanoparticle-treated groups, with either intratracheal instillation or inhalation during the observation period. These results indicated that NiO, ZnO and CeO_2_ nanoparticles generate significant amounts of free radicals, and oxidative stress may be responsible for the lung injury caused by these nanoparticles. In addition, both intratracheal instillation and inhalation exposure induced similar tendencies of oxidative DNA damage with these nanoparticles.

## Introduction

Nanoparticles (NPs) are widely used in various commercial products, due to their unique physicochemical properties. However, the safety issues for humans exposed to nanoparticles are now attracting more attention. Recent studies indicated that some nanoparticles induce oxidative stress and increase the risk of cancer.^(1–4)^ The highly specific surface areas of nanoparticles are very reactive, and could lead to an increase in the production of reactive oxygen species (ROS). As a result, NPs cause damage to human and animal cells.^(5)^ Oxidative damage of DNA, proteins and/or lipids can induce chromosome instability, mutations and modulations of cell growth that may result in disease. Oxidative stress is considered to elevate the risk of lifestyle-related diseases, such as diabetes and cancer.^(6)^ It has been proposed that 8-hydroxydeoxyguanosine (8-OHdG), a biomarker of oxidative DNA damage, is formed abundantly by oxidative stress and causes mutations.^(7,8)^ Our previous studies suggested that oxidative stress might be a major factor underlying the pulmonary toxicity caused by CeO_2_, TiO_2_, NiO and ZnO nanoparticles.^(9–12)^ In this study, we measured the 8-OHdG levels to evaluate the oxidative DNA damage elicited by the administration of NPs to rat lungs. For the accurate risk assessment of NPs, an appropriate administration technique is required. Although the most authentic simulation model would be the inhalation method, because it most closely simulates the actual exposure to humans, other appropriate models should be assessed. As a simple and quick treatment, intratracheal instillation may be useful. However, only few reports have compared the induction of oxidative DNA damage *in vivo* caused by nanoparticle exposure via intratracheal instillation and inhalation. In this study, we compared the oxidative DNA damage in lung tissue caused by exposure to TiO_2_, NiO, ZnO and CeO_2_ nanoparticles, administered by intratracheal instillation or inhalation.

## Materials and Methods

### Rat lung tissue

Tissues frozen at −80°C, obtained in our previous studies,^(9–12)^ were analyzed. The animals and the methods of NP treatment are briefly described as follows. Commercially purchased TiO_2_ (rutile, MT-150AW, Teyka Co. Ltd., Osaka, Japan), NiO (US 3355, US Research Nanomaterials, Houston, TX), ZnO (Sigma-Aldrich Co. LLC., Tokyo, Japan, 51 wt.% ZnO), and CeO_2_ (Wako Pure Chemicals, Ltd., Japan) nanoparticles were used. The characterization of the nanoparticles was reported previously. The details of the nanoparticle dispersion for intratracheal instillation and inhalation were described by Morimoto *et al.*^(10)^ Male Fischer 344 rats (9–11 weeks old) were purchased from Charles River Laboratories International, Inc. (Japan). The animals were fed a commercial diet and water *ad libitum*, and were kept in the laboratory of the Animal Research Center at the University of Occupational and Environmental Health for 2 weeks. All of the animal experimental procedures and handing methods were performed in accordance with the guidelines described in the Japanese Guide for the Care and Use of Laboratory Animals, as approved by the Animal Care and Use Committee, University of Occupational and Environmental Health, Japan. The nanoparticles (0.2 mg and 1.0 mg) were suspended in 0.4 ml distilled water. The average particle diameters, measured by dynamic light scattering, were TiO_2_: 44.9 nm; NiO: 59.7 nm; ZnO: 33 nm; and CeO_2_: 10 nm. Each nanoparticle suspension was administered to rats (12-week-old) by a single intratracheal instillation. For the negative control groups, 0.4 ml of distilled water was instilled. The details of the experimental setup and the conditions for the inhalation study were described previously.^(13,14)^ The high and low dose chambers were used in this study. The concentrations of the particles were TiO_2_: 0.50 ± 0.26 mg/m^3^ and 1.84 ± 0.74 mg/m^3^; NiO: 0.32 ± 0.07 mg/m^3^ and 1.65 ± 0.20 mg/m^3^; ZnO: 2.11 ± 0.45 mg/m^3^ and 10.40 ± 1.39 mg/m^3^; CeO_2_: 2.09 ± 0.29 mg/m^3^ and 10.20 ± 1.38 mg/m^3^, respectively. The particle size distributions had two peaks, at around 30 and 200 nm for TiO_2_ and 30 and 100 nm for NiO. The geometric mean diameters of the aerosol particles in the high dose chamber were 148 nm for ZnO and 110 nm for CeO_2_. The 10-week-old rats were exposed to nanoparticles in a whole-body exposure chamber (volume, 0.52 m^3^) for 6 h/day and 5 days/week for 4 weeks. In the same air-conditioned room, the control rats were exposed to clean air in an equivalently sized chamber. The rats were dissected at 3 days, 1 month, 3 months and 6 months after exposure to nanoparticles by intratracheal instillation or inhalation. The lung tissue was promptly removed and stored at −80°C until analyzed.

### Analysis of 8-OHdG in lung DNA

The lung nuclear DNA was extracted from 100 mg of tissue from the right upper lobe. The 8-OHdG levels in the DNA were measured according to our previous report,^(15)^ with a slight modification. A pretreatment filter (EKICRODISC, Acro LC3CR, Nihon Pall Ltd., Tokyo, Japan) was used to filter the digested nucleoside solution. The filtrate was kept at −80°C until just before analysis. A 40 µl aliquot of the filtrate was injected into an HPLC column (Capcell Pak C18 MGII, 3 µm, 4.6 × (100 mm + 150 mm: series-connected), Shiseido Fine Chemicals, Tokyo, Japan) equipped with an electrochemical detector (ECD-300, Eicom Co., Kyoto, Japan). The flow rate was 0.7 ml/min. The 8-OHdG values in the DNA were calculated as the number of 8-OHdG per 10^6^ deoxyguanosine (dG).

### Statistical analysis

All data were statistically analyzed by the analysis of variance (ANOVA) to determine the individual differences, using a computer statistical package (SPSS, SPSS Inc., Chicago, IL). The data are expressed as the mean ± SD. Statistical significance was assessed as *p<0.05, **р<0.01.

## Results

### Analysis of 8-OHdG in lung DNA with intratracheal instillation exposure

**Fig. 1:**
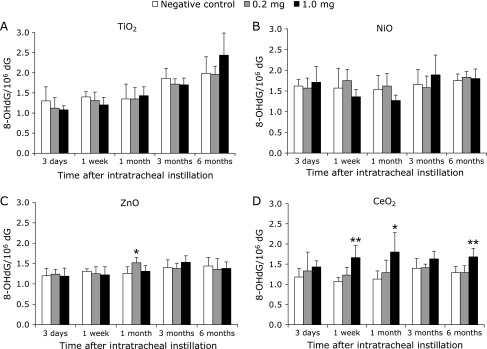
Effects of nanoparticle exposure on the levels of 8-OHdG in lung DNA after intratracheal instillation. The levels of 8-OHdG in lung DNA upon TiO_2_ nanoparticle exposure (A), NiO nanoparticle exposure (B), ZnO nanoparticle exposure (C), and CeO_2_ nanoparticle exposure (D). 8-OHdG levels were measured with an HPLC-EC detector. Values are mean ± SD (*n* = 5). Significant differences versus control group are indicated in the figure (ANOVA). *p<0.05, **p<0.01.

The 8-OHdG levels in pulmonary DNA, following the intratracheal instillation of nanoparticles, are shown in Fig. 1. No significant differences were found between the TiO_2_ and NiO nanoparticle exposure groups and the controls (Fig. 1A, B) during the experimental periods. As compared to the control group, the 8-OHdG level was only significantly increased in the 0.2 mg ZnO nanoparticle group at 1 month after intratracheal instillation exposure (Fig. 1C). In contrast, in the 1.0 mg CeO_2_ nanoparticle intratracheal instillation group, the 8-OHdG levels were significantly and persistently higher than those in the control group throughout the experimental period (Fig. 1D).

### Analysis of 8-OHdG in lung DNA with inhalation exposure

**Fig. 2:**
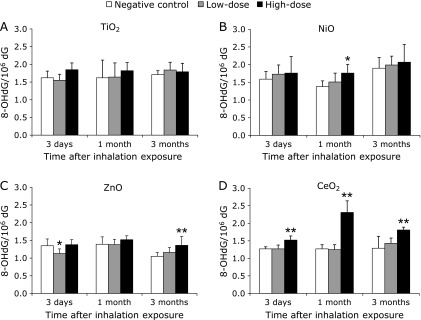
Effects of nanoparticle exposure on the levels of 8-OHdG in lung DNA after inhalation. The levels of 8-OHdG in lung DNA upon TiO_2_ nanoparticle exposure (A), NiO nanoparticle exposure (B), ZnO nanoparticle exposure (C), and CeO_2_ nanoparticle exposure (D). 8-OHdG levels were measured with an HPLC-EC detector. Values are mean ± SD (*n* = 5). Significant differences versus control group are indicated in the figure (ANOVA). *p<0.05, **p<0.01.

There were no significant differences in the 8-OHdG levels in lung DNA between the TiO_2_ nanoparticle inhalation groups and the controls (Fig. 2A). In contrast, the 8-OHdG levels in pulmonary DNA were increased in a dose-dependent manner by NiO inhalation at all time points (Fig. 2B). In the high-dose ZnO exposure group, the 8-OHdG levels in the lung DNA were significantly increased at 3 months after inhalation exposure (Fig. 2C). The 8-OHdG levels were significantly and persistently higher than those of the controls in the high-dose groups of CeO_2_ throughout the experimental period (Fig. 2D).

The trends of 8-OHdG induction by the TiO_2_, ZnO and CeO_2_ nanoparticles in rat pulmonary DNA were qualitatively similar for both the intratracheal instillation and inhalation exposure methods. However, the time courses leading to the significant increase of the 8-OHdG levels were different in the ZnO treatment groups. The significant increase in the 8-OHdG levels by inhalation (3 months) occurred later than that induced by intratracheal instillation (1 month).

## Discussion

In this study, through measurements of the 8-OHdG levels in rat lung DNA, we assessed the oxidative DNA damage induced by four metallic oxide nanoparticles (TiO_2_, NiO, ZnO and CeO_2_) administered by intratracheal instillation and inhalation. Many studies have reported that oxidative stress is induced by exposure to metal nanoparticles.^(1,4,16,17)^ A few studies have shown the induction of oxidative DNA damage by nanoparticle exposure, by measuring the 8-OHdG levels *in vitro* or *in vivo*.^(18,19)^ However, to the best of our knowledge, no study has compared the 8-OHdG levels in lung DNA after exposure to nanoparticles by intratracheal instillation and inhalation. We previously reported^(9–12)^ that the levels of heme oxygenase (HO-1), an oxidative stress marker, in bronchoalveolar lavage fluid (BALF) were not affected or transiently increased by the intratracheal instillation and inhalation of TiO_2_, respectively. In the NiO treatment via these two exposure methods, the HO-1 levels were significantly increased with exposure to both the low and high concentrations during the experimental period. However, in the present study, the 8-OHdG levels were only increased in a dose-dependent manner with the inhalation of NiO, but not with the intratracheal instillation. Even though measurements of other oxidative stress markers may help to clarify the mechanisms underlying the adverse health effects of metal oxide nanoparticles, both HO-1 and 8-OHdG are typical oxidative stress markers. HO-1 is an enzyme that protects organisms from oxidative damage, and is induced by oxidative stress. In contrast, 8-OHdG is DNA damage produced by oxidative stress. Therefore, the 8-OHdG levels may not be increased upon the induction of HO-1. For evaluating adverse health effects associated with DNA damage, such as carcinogenesis and mutagenesis, measurements of 8-OHdG are probably useful. In our previous reports,^(9–11)^ nanoparticle-induced inflammation was demonstrated by measuring inflammation markers, such as neutrophil counts and chemokines. The measurement of 8-nitroguanine, a nitrative DNA lesion formed under inflammatory conditions,^(20)^ may provide further information about nanoparticle toxicology. Regarding ZnO, the increases in the 8-OHdG levels in lung DNA were observed 1 month after intratracheal instillation and 3 months after inhalation. Driscoll *et al.*^(21,22)^ considered the types of responses to be similar with intratracheal instillation and inhalation; however, the time courses and strengths of the responses were different. Generally, the biological responses induced by intratracheal instillation are faster and stronger than those induced by inhalation. In the CeO_2_ nanoparticle groups, a persistent increase in the 8-OHdG levels in lung DNA was observed in the high concentration groups with both intratracheal instillation and inhalation. The high 8-OHdG levels, persisting long after the administration, may be related to the long period of nanoparticle retention in the lung. In fact, nanoparticles were observed by transmission electron microscopy in the lung tissue at 3 months after administration.^(11)^ The CeO_2_ nanoparticles induced the strongest oxidative DNA damage in the rat lung, in this intratracheal instillation study. Although the underlying reason is unclear, it may be related to nanoparticle clearance from the lung, antioxidant depression, or depression of DNA repair enzymes.^(23)^ Further studies are necessary to elucidate the mechanism underlying the adverse health effects of metal oxide nanoparticles.

Overall, the 8-OHdG levels in the lung DNA showed similar trends by exposure to these nanoparticles between the intratracheal instillation and inhalation methods. In our previous study,^(12)^ a similar amount of NiO induced comparable pulmonary oxidative stress with both the intratracheal instillation and inhalation administration methods. In our experimental conditions, the initial lung burdens by the intratracheal instillations of NiO and TiO_2_ (0.2 mg/rat) were approximately the same as the high inhalation doses. In the cases of ZnO and CeO_2_, the initial lung burdens of the low- and high-concentration groups in the intratracheal instillation were similar to those of the inhalation experiments of the low- and high-concentration groups.^(9–11)^ Considering the difficulty of the inhalation procedure, the intratracheal instillation method may be useful for estimating the adverse health effects of nanoparticles, especially in screening assays.

In summary, NiO, ZnO and CeO_2_ nanoparticles generated significant oxidative DNA damage, which may be responsible for the lung injury caused by these nanoparticles. Furthermore, the intratracheal instillation and inhalation exposure methods exhibited similar tendencies in the induction of oxidative DNA damage.

## Abbreviations

BALF: bronchoalveolar lavage fluid
NPs: nanoparticles
8-OHdG: 8-hydroxydeoxyguanosine

## References

[1] Fukui H, Horie M, Endoh S (2012). Association of zinc ion release and oxidative stress induced by intratracheal instillation of ZnO nanoparticles to rat lung. Chem Biol Interact.

[2] Pujalté I, Passagne I, Brouillaud B (2011). Cytotoxicity and oxidative stress induced by different metallic nanoparticles on human kidney cells. Part Fibre Toxicol.

[3] Kim IS, Baek M, Choi SJ (2010). Comparative cytotoxicity of Al_2_O_3_, CeO_2_, TiO_2_ and ZnO nanoparticles to human lung cells. J Nanosci Nanotechnol.

[4] Siddiqui MA, Ahamed M, Ahmad J (2012). Nickel oxide nanoparticles induce cytotoxicity, oxidative stress and apoptosis in cultured human cells that is abrogated by the dietary antioxidant curcumin. Food Chem Toxicol.

[5] Khalili Fard J, Jafari S, Eghbal MA (2015). A review of molecular mechanisms involved in toxicity of nanoparticles. Adv Pharm Bull.

[6] Kohen R, Nyska A (2002). Oxidation of biological systems: oxidative stress phenomena, antioxidants, redox reactions, and methods for their quantification. Toxicol Pathol.

[7] Kasai H (2002). Chemistry-based studies on oxidative DNA damage: formation, repair, and mutagenesis. Free Radic Biol Med.

[8] Kasai H, Kawai K, Jurga S, Erdmann VA, Barciszewski J (2016). 8-Hydroxyguanine, an oxidative DNA and RNA modification. Modified Nucleic Acids in Biology and Medicine.

[9] Morimoto Y Izumi H Yoshiura Y Evaluation of pulmonary toxicity of zinc oxide nanoparticles following inhalation and intratracheal instillation Int J Mol Sci 2016 17 pii: E1241. DOI: 10.3390/ijms17081241 10.3390/ijms17081241 PMC5000639 27490535

[10] Morimoto Y, Izumi H, Yoshiura Y (2016). Comparison of pulmonary inflammatory responses following intratracheal instillation and inhalation of nanoparticles. Nanotoxicology.

[11] Morimoto Y, Izumi H, Yoshiura Y (2015). Pulmonary toxicity of well-dispersed cerium oxide nanoparticles following intratracheal instillation and inhalation. J Nanopart Res.

[12] Horie M, Yoshiura Y, Izumi H (2016). Comparison of the pulmonary oxidative stress caused by intratracheal instillation and inhalation of NiO nanoparticles when equivalent amounts of NiO are retained in the lung. Antioxidants (Basel)..

[13] Kubo M, Nakaoka A, Morimoto K (2014). Aerosol generation by a spray-drying technique under coulomb explosion and rapid evaporation for the preparation of aerosol particles for inhalation tests. Aerosol Sci Tech.

[14] Shimada M, Wang WN, Okuyama K (2009). Development and evaluation of an aerosol generation and supplying system for inhalation experiments of manufactured nanoparticles. Environ Sci Technol.

[15] Kawai K, Li YS, Kasai H (2007). Accurate measurement of 8-OH-dG and 8-OH-Gua in mouse DNA, urine and serum: effects of X-ray irradiation. Gene Environ.

[16] Xia T, Kovochich M, Liong M (2008). Comparison of the mechanism of toxicity of zinc oxide and cerium oxide nanoparticles based on dissolution and oxidative stress properties. ACS Nano.

[17] Huerta-Garcia E, Pérez-Arizti JA, Márquez-Ramirez SG (2014). Titanium dioxide nanoparticles induce strong oxidative stress and mitochondrial damage in glial cells. Free Radic Biol Med.

[18] Bhattacharya K, Davoren M, Boertz J, Schins RP, Hoffmann E, Dopp E (2009). Titanium dioxide nanoparticles induce oxidative stress and DNA-adduct formation but not DNA-breakage in human lung cells. Part Fibre Toxicol.

[19] Song MF, Li YS, Kasai H, Kawai K (2012). Metal nanoparticle-induced micronuclei and oxidative DNA damage in mice. J Clin Biochem Nutr.

[20] Hiraku Y (2010). Formation of 8-nitroguanine, a nitrative DNA lesion, in inflammation-related carcinogenesis and its significance. Environ Health Prev Med.

[21] Driscoll KE, Lindenschmidt RC, Maurer JK, Higgins JM, Ridder G (1990). Pulmonary response to silica or titanium dioxide: inflammatory cells, alveolar macrophage-derived cytokines, and histopathology. Am J Respir Cell Mol Biol.

[22] Driscoll KE, Lindenschmidt RC, Maurer JK, Perkins L, Perkins M, Higgins J (1991). Pulmonary response to inhaled silica or titanium dioxide. Toxicol Appl Pharmacol.

[23] Hirano T, Yamaguchi Y, Kasai H (1997). Inhibition of 8-hydroxyguanine repair in testes after administration of cadmium chloride to GSH-depleted rats. Toxicol Appl Pharmacol.

